# Is There an Oncological Benefit of Performing Bilateral Pelvic Lymph Node Dissection in Patients with Penile Cancer and Inguinal Lymph Node Metastasis?

**DOI:** 10.3390/jcm10040754

**Published:** 2021-02-13

**Authors:** Rodrigo Suarez-Ibarrola, Mario Basulto-Martinez, August Sigle, Mohammad Abufaraj, Christian Gratzke, Arkadiusz Miernik

**Affiliations:** 1 Department of Urology, Faculty of Medicine, University of Freiburg—Medical Centre, 79106 Freiburg, Germany; august.sigle@uniklinik-freiburg.de (A.S.); christian.gratzke@uniklinik-freiburg.de (C.G.); arkadiusz.miernik@uniklinik-freiburg.de (A.M.); 2 Department of Urology, Hospital Regional de Alta Especialidad de la Peninsula de Yucatán, Merida 97133, Mexico; basultourologia@gmail.com; 3 Division of Urology, Department of Special Surgery, Jordan University Hospital, The University of Jordan, Amman 11942, Jordan; mabufaraj@ju.edu.jo

**Keywords:** penile cancer, penile carcinoma, ILND, inguinal lymph node dissection, PLND, pelvic lymph node dissection

## Abstract

We aim to review the literature for studies investigating the oncological outcomes of patients with penile cancer (PC) undergoing bilateral pelvic lymph node dissection (PLND) in the presence of inguinal lymph node metastasis (LNM) who are at risk of harboring pelvic metastasis. A search of English language literature was performed using the PubMed-MEDLINE database up to 3 December 2020 to identify articles addressing bilateral PLND in PC patients. Eight articles investigating bilateral PLND met our inclusion criteria. Patients with pelvic LNM have a dismal prognosis and, therefore, PLND has an important role in both the staging and treatment of PC patients. Ipsilateral PLND is recommended in the presence of ≥2 positive inguinal nodes and/or extranodal extension (ENE). Significant survival improvements were observed with a higher pelvic lymph node yield, in patients with pN2 disease, and in men treated with bilateral PLND as opposed to ipsilateral PLND. Nevertheless, the role of bilateral PLND for unilateral inguinal LNM remains unclear. Although the EAU guidelines state that pelvic nodal disease does not occur without ipsilateral inguinal LNM, metastatic spread from one inguinal side to the contralateral pelvic side has been reported in a number of studies. Further studies are needed to clarify the disseminative pattern of LNM, in order to establish PLND templates according to patients’ risk profiles and to investigate the benefit of performing bilateral PLND for unilateral inguinal disease.

## 1. Introduction

The development of lymph node metastases (LNM) in penile cancer (PC) follows the anatomical loco-regional drainage route and is characterized by a stepwise disseminative pattern [1]. The presence and extent of LNM is the single most important prognostic factor in determining long-term survival in men with invasive PC [2]. The European Association of Urology (EAU) and the National Comprehensive Cancer Network (NCCN) guidelines recommend performing an inguinal lymph node dissection (ILND) in patients with a Union for International Cancer Control TNM stage ≥ T1bG2 [3,4]. The number, diameter, and extracapsular nodal extension (ENE) of inguinal metastases have been established as independent risk factors for pelvic lymph node involvement, which carries a poor prognosis. Furthermore, the proportion of pelvic LNM was shown to increase from 0% to 57.1% in cases with no risk factors and when all three are present, respectively [5]. Therefore, ipsilateral pelvic lymph node dissection (PLND) is recommended as an adjunct to ILND in patients with ≥2 inguinal LNM on one side and/or ENE for proper lymphatic assessment and staging [3].

Nevertheless, controversy exists regarding the therapeutic role of PLND since the proportion of patients with pelvic LNM among those with inguinal LNM is highly variable, and it has been reported that only a fraction of patients with pelvic LNM may benefit from PLND alone [6]. Other studies suggest that PLND has a curative role only in patients with a single microscopic focus in the pelvic specimen [7]. A further polemic is whether PLND should be performed ipsilaterally only or bilaterally in patients with unilateral inguinal LNM since crossover from inguinal to contralateral pelvic nodes has not been well studied [8]. The rarity of PC and the lack of randomized studies preclude high-level evidence recommendations in the management of pelvic lymph nodes, and its benefit in low-risk patients remains to be confirmed.

This study aimed to retrieve articles addressing the oncological outcomes of patients with penile cancer (PC) undergoing bilateral PLND for inguinal LNM at risk of harboring pelvic LNM.

## 2. Materials and Methods

### 2.1. Search Strategy

A search was conducted using the PubMed-MEDLINE database up to 3 December 2020 with the following search string: (((“Penile Neoplasms”[MeSH]) OR (penile cancer)) OR (penile carcinoma)) AND (((((lymph node excision [MeSH]) OR (lymph node dissection)) OR (lymphadenectomy)) OR (pelvic lymph node dissection)) OR (pelvic lymphadenectomy)).

**Figure 1:**
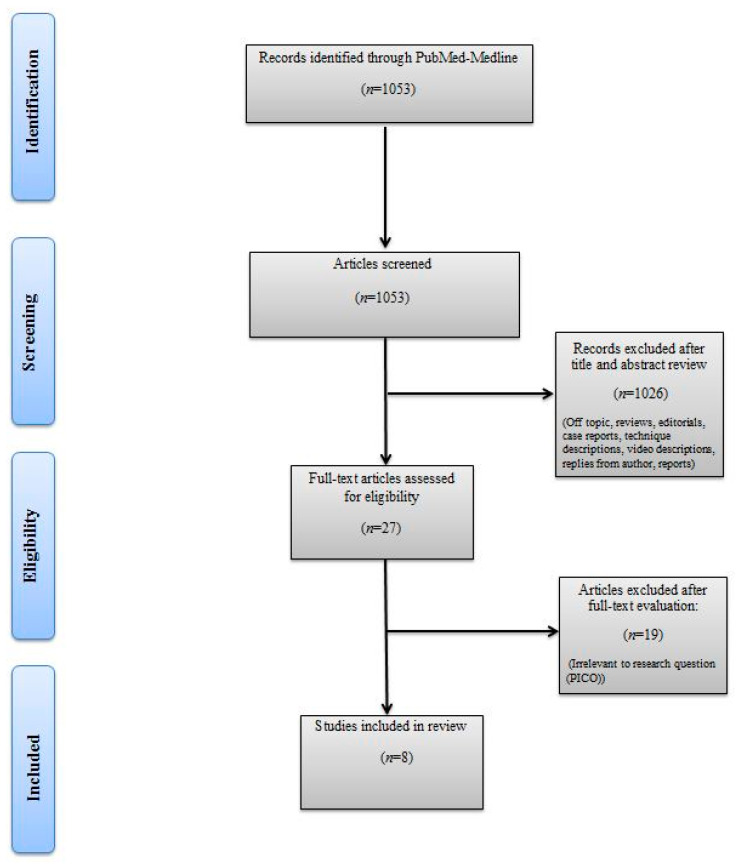
Preferred Reporting Items for Systematic Reviews and Meta-analyses (PRISMA) flow chart.

The titles and abstracts of identified articles were retrieved for evaluation. The full text of potentially eligible articles was independently screened against the study selection criteria by two independent reviewers (M.B. and R.S.). Disagreement was resolved by discussion; if no agreement was reached, a third independent party acted as an arbiter (A.M). Figure 1 shows the Preferred Reporting Items for Systematic Reviews and Meta-analyses (PRISMA) flow diagram detailing the search strategy and identification of studies used in the evidence synthesis.

### 2.2. Inclusion and Exclusion Criteria

According to the PRISMA statements, the PICOS: Population (P), Intervention (I), Comparator (C), Outcomes (O), and Study design (S), approach was used to specify the eligibility criteria. Therefore, studies were considered eligible if PC patients (P) were managed by bilateral PLND for inguinal LNM (I) as a single-arm or compared to patients in whom unilateral PLND or no PLND was performed (C) and if their oncological outcomes (O) were assessed in retrospective or prospective studies (S). After article selection and according to the eligibility criteria, the following types of studies were excluded: articles not describing bilateral PLND, articles not written in English, review articles, conference abstracts, and case reports.

### 2.3. Data Extraction and Analysis

A data extraction form was developed to collect information on author and journal, study design and period, purpose of investigation, number of patients included, patient age, type of intervention, TNM stage, tumor differentiation and lymphovascular invasion, ENE, PLND template, complications, local, regional, or systemic recurrences, neo/adjuvant treatment, follow-up period, lymph node yield and positivity, and survival analysis. Two reviewers independently extracted data for further assessment of qualitative and quantitative evidence synthesis. Descriptive statistics were used for baseline data and a narrative synthesis of the evidence was performed.

## 3. Results

Table 1 summarizes the demographic, baseline characteristics, and perioperative data of studies which included patients treated with bilateral PLND for PC. Eight studies were included that reported on the oncological outcomes of bilateral PLND in 619 patients with PC who underwent PLND, of which 420 were bilateral. All eight studies reported the anatomical boundaries of the PLND templates, of which three described the surgical technique for PLND in detail [7,9,10]. Seven studies were of a retrospective nature, while one study performed ilioinguinal lymph node dissections prospectively [10]. Table 2 summarizes the inguinal and pelvic lymph node status and survival analysis of patients treated for PC.

### 3.1. Pelvic Lymph Node Yield Impacts Survival

Chipollini et al. performed PLND in 198 chemonaïve patients, of which 106 (53.5%) were bilateral dissections. The median number of lymph nodes (LN) and the median number of positive LN were 13 (8–19) and 2 (1–4), respectively. The survival analysis revealed that patients with a lymph node yield of ≥9 LN had better 5-year disease-specific survival (DSS) (64.2% vs. 47.2%), overall survival (OS) (60.3% vs. 39.8%), and recurrence-free survival (RFS) (60.3% vs. 43.2%). Moreover, a lymph node yield of ≥9 was found to be a predictor for RFS after adjustment for baseline characteristics. Although a distinction of DSS, OS, and RFS differences in patients that underwent unilateral as opposed to bilateral PLND was not undertaken, this study established an LN threshold that may be more readily obtained with bilateral dissections and that may provide a benefit in patients harboring pelvic LNM [11].

### 3.2. Prophylactic PLND

Djajadiningrat et al. prophylactically treated 79 chemonaïve PC patients with PLND, of which 23 (29%) were bilateral, when ≥2 positive inguinal LN or ENE were found. Tumor-positive pelvic nodes were found in 19 (24%) patients, and both inguinal ENE and ≥2 positive inguinal LN were identified as predictors of pelvic nodal involvement. A plausible explanation for ENE predicting pelvic LNM may lie in tumors’ aggressiveness and ability to penetrate the lymph node capsule. Moreover, patients with positive pelvic LNM had worse 5-year DSS compared to patients without pelvic LNM (17% vs. 62%). There was no comparison of outcomes between ipsilateral and bilateral PLND [7].

### 3.3. Bilateral PLND vs. No-PLND

In a multicenter study by Li et al., 69 patients underwent bilateral PLND and were compared to 121 patients in whom PLND was spared. Among the PLND group, 16 patients had unilateral and five patients had bilateral pelvic LNM, and no patient had crossover metastatic spread from one inguinal side to the contralateral pelvic side. The PLND group did not demonstrate higher 1- and 3-year DSS rates than the no-PLND group (65.7% and 39.0% vs. 65.4% and 39.6%, *p* = 0.796). However, propensity score matching among pN2 patients showed higher DSS in patients who underwent PLND compared to those who did not (83.3% and 83.3% vs. 69.0% and 50.2%, *p* = 0.030). Based on their analyses, a significant benefit was found for a subset of pN2 cases, while pN3 patients may not benefit from bilateral PLND [12].

### 3.4. Bilateral vs. Unilateral PLND

In another multicentric study, Zargar-Shoshtari et al. investigated criteria predicting bilateral pelvic LNM in patients with pathologically confirmed inguinal nodes. In a cohort of 140 patients, 83 had both bilateral inguinal and pelvic LNM, and bilateral PLND was performed in 64 (77%) patients. Of note, 27 (32.5%) patients received neoadjuvant chemotherapy (NAC). The presence of ≥4 positive inguinal nodes had a sensitivity of 95% and an area under the curve (AUC) of 0.76 for predicting bilateral pelvic LNM, which was present in only one patient with <4 inguinal LNM. In a separate analysis which excluded patients who received NAC, ≥4 inguinal LNM was still the strongest predictor for bilateral disease; however, it did not reach statistical significance. The rate of regional failure was higher for patients who only had ipsilateral PLND compared to bilateral PLND (47% at 5.53 vs. 20% at 12.8 months, *p* = 0.02). Additionally, there was a trend for improved OS in men treated with bilateral PLND compared to unilateral (11.8 vs. 10.9 mo., *p* = 0.10), and OS was significantly lower with ≥4 positive inguinal LNM (8.7 vs. 15.4 months, *p* = 0.04) [13].

In a subsequent multicentric study, the same authors assessed the oncological outcomes of patients treated with bilateral PLND in the presence of unilateral pelvic LNM. In 51 patients with unilateral inguinal LNM and positive pelvic nodes, 38 (75%) had ipsilateral and 13 (25%) bilateral PLND. In the 13 patients with bilateral PLND, metastases were only found in ipsilateral pelvic nodes. It is important to note that 86% of the cohort received preoperative and postoperative chemotherapy, radiotherapy, or both, and a higher proportion of adjuvant radiotherapy was utilized in the unilateral PLND patients (50% vs. 8%, *p* = 0.01). Despite this, the median OS was significantly longer in bilateral PLND patients compared to unilateral (21.7 vs. 13.1 mo., *p* = 0.05) at a median 13.3 months follow-up. Cancer-specific survival (CSS) was statistically similar in bilateral and ipsilateral PLND, but regression analysis showed that bilateral PLND, adjusted for additional therapies and presence of multiple pelvic LNM, improved CSS [14].

### 3.5. Predicting the Disseminative Pattern of Pelvic LNM

Zhu et al. evaluated the value of computerized tomography, Cloquet’s node, and inguinal LN disease burden in predicting pelvic LNM. A cohort of 73 patients underwent bilateral ILND, of which 33 had inguinal LNM and 16 pelvic LNM. Cloquet’s node had a sensitivity of 30% and a specificity of 94.1% in pelvic CT-negative groin basins. The number of positive inguinal LNs, lymph node ratio (number of positive LNs/total number removed), ENE, and p53 expression were significantly associated with pelvic LNM. Prognostic factors for pelvic LNM were ≥3 enlarged inguinal LNs in preoperative CT imaging and lymph node size of ≥3.5 cm as the maximum diameter. Importantly, no patient developed pelvic LNM in the absence of inguinal LNM and contralateral pelvic LNM was not observed in unilateral inguinal LNM [9].

Zhu et al. further investigated the disseminative pattern of LNM in 46 chemonaïve patients that underwent bilateral ILND. In those with ≥1 positive inguinal LNM, bilateral iliac LND was performed. Among these patients, 21 had unilateral inguinal LNM and three bilateral LNM. The positive and negative predictive values of Cloquet’s node for predicting iliac LNM were 80% and 86%, respectively. Of 48 ILND, 21 had no LNM, and in the 21 groin basins, ilioinguinal LND did not reveal iliac LNM in the absence of inguinal LN involvement [10].

### 3.6. Lymph Node Mapping

Recently, Yao et al. provided an accurate dissemination map of LNM and aimed to determine the extent of PLND. One hundred and twenty-eight chemo-radio-naive patients received bilateral ILND, 111 received bilateral PLND, and 17 received unilateral PLND. Pelvic LNM was present in 57 (44.5%) patients and bilateral LNM was found in 13 patients. The median number of pelvic LNs removed was 18 (IQR: 10–30). All 57 patients with PLNM had inguinal LNM. As opposed to other studies where crossover was not found [9,10,12], two patients with unilateral inguinal LNM had bilateral PLNM, suggesting crossover from inguinal nodes to contralateral pelvic nodes. Lopes et al. similarly documented metastasis to the pelvic nodes with no inguinal involvement [15].

OS was significantly longer in patients that underwent bilateral PLND than those with unilateral (30 vs. 18 mo., *p* = 0.004). Among the 57 patients with pelvic LNM, the estimated median OS was higher in the bilateral than in the unilateral group (16 vs. 11 mo., *p* = 0.07). Moreover, the prevalence of pelvic LNM was similar in patients with ENE and without ENE (42.9% vs. 45.8%, *p* = 0.74), which questions the validity of ENE for predicting pelvic LNM, as has been reported by a multitude of studies [8].

## 4. Conclusions

There is a paucity of data on the disseminative pattern of pelvic LNM in patients with PC. Moreover, there are scarce data comparing the outcomes of patients treated with bilateral vs. unilateral PLND in the presence of unilateral inguinal disease. Although the EAU guidelines state that crossover metastatic spread from one groin to the contralateral pelvis has never been reported [3], a number of studies have found metastases to pelvic nodes without ipsilateral inguinal involvement [8,12,15]. Interestingly, despite the rarely found crossover, data gathered in the current review suggest that bilateral PLND is beneficial, but the ideal candidate is yet to be determined.

Among the justifications for performing bilateral PLND is the improved survival observed compared to patients treated unilaterally, and particularly in the pN2 subset of patients, both of which may correlate with the treatment of occult micrometastatic disease. Moreover, a possible explanation for the observation of minimal crossover metastatic spread to contralateral pelvic LNs in patients with unilateral inguinal LNM may be disease underestimation even when invasive nodal staging is performed.

Despite patients with invasive or high-grade PC seemingly benefiting from undergoing extensive lymph node dissection, both ILND and PLND are characteristically associated with considerable physical and psychological morbidity. However, only a single study reported data on postoperative complications in patients that underwent bilateral PLND, where 14 patients (18%) had some type of complication, 9 of these wound-related and 5 non-wound-related such as pneumonia, delirium, and ileus [7]. Modern series report complication rates of 42–57% for all patients undergoing ILND, the most common being wound necrosis and dehiscence, lymphocele, lymphoedema, and venous thromboembolism [16]. Several strategies have been recommended to prevent adverse effects of ILND such as prophylactic antibiotics until drain removal, early ambulation, debridement of non-viable skin, meticulous control of lymphatics, preservation of the saphenous vein, and antiembolism stockings, none of which have been studied in prospective trials [17]. Contemporary minimally invasive management strategies for LN involvement aim to minimize the morbidity associated with traditional radical inguinal and pelvic lymphadenectomy through appropriate risk stratification whilst optimizing oncological outcomes.

Due to inherent methodological limitations, false-negative rates of inguinal and pelvic lymphadenectomy remain unknown. The implementation of new imaging modalities such as ^18^FDG-PET/CT into clinical workflows may improve the diagnostic accuracy for the detection of LNM [18,19,20]. A recent Danish study by Jakobsen et al. found ^18^FDG-PET/CT to have a groin false-negative rate of 2.1% and a sensitivity of 85.4% per patient for assessing inguinal lymph node metastasis and distant metastasis compared to 47.5% for the conventional CT scan [21]. Graafland et al. reported a sensitivity of 91% and a specificity of 100% with PET/CT for pelvic staging [6]. In contrast, for dynamic sentinel-node biopsy, false-negative rates of up to 12–15% have been reported [22]. Consequently, these approaches might help to address challenges in adequately identifying patients that might benefit from bilateral PLND and assist in treatment decision making.

However, specific bilateral PLND indications and patient selection need to be better defined. Furthermore, studies are needed addressing the value of the LN yield as a surrogate of survival. Since retrospective studies cannot establish causality properly, ongoing prospective randomized trials, such as the International Penile Advanced Cancer Trial (InPACT), may help clarify the role of PLND and its integration with multimodal therapies. Studies are warranted to determine the benefit of bilateral PLND in patients with unilateral inguinal LNM suspected of harboring contralateral metastatic disease. This implies studies that help clarify the disseminative pattern of metastatic spread and the limits of dissection in a precise and personalized manner.

The clinical heterogeneity within studies and the limitations of the study need to be acknowledged. Firstly, the search strategy was not systematically performed, and therefore the acquisition of all bilateral PLND studies cannot be confirmed. Secondly, there was not enough information to propose a meta-analysis given the little convergence within studies, which would incur in a high risk of outcome measurement bias. Not all patients that underwent bilateral PLND had the same tumor stage, nor did they receive the same treatment regimen or LND templates, which precludes the comparison of outcomes. The different PLND templates, aims/outcomes of studies, the low patient number, interference of neo/adjuvant treatments, and the retrospective nature of studies contribute to the heterogeneity of data.

Although pelvic and inguinal LNM dissemination patterns are predominantly reported following the anatomical staged pathway, exceptions exist and further efforts and larger cohorts are needed to deepen the understanding of LNM spread, as they may provide insights for directing LND strategies. Moreover, data suggest that preoperative imaging and staging may play a role in identifying pelvic LN involvement. Therefore, further research in this field may contribute to better patient selection for either unilateral or bilateral PLND, while weighing the oncological benefits, perioperative complications, and patients’ quality of life.

## Figures and Tables

**Table 1:** Summary of studies including patients treated with bilateral pelvic lymph node dissection for penile cancer.

Author	Study Design and Period	Study Purpose	Patients (*n*)	Median Age (IQR)	TNM Stage *n* (%)	Differentiation *n* (%)	PLND Template	Lymphovascular Invasion	Extracapsular Nodal Extension (ENE)	Neo/Adjuvant Treatment *n* (%)	Local/Regional / Systemic Recurrence *n* (%)	Mean Follow-Up Months (IQR)
Zhu et al., Onkologie 2008 [9]	Retrospective. 1990–2005	Predicting PLNM comparing CT, Cloquet’s node, and ILN burden	73 patients underwent bilateral ILND and 10 bilateral PLND	NA	NA	Total G1-2: 10 G3: 23 Present in PLNM G1-2: 2 G3: 14	Common iliac, external iliac, and internal iliac	NA	No-PLNM Absent: 17 Present: 0 PLNM Absent: 11 Present: 5	NA	NA	28 (8–172) median, range
Zhu et al., J Urol 2009 [10]	Prospective single-center. 2006–2008	Disseminative pattern of PLNM	46 total 92 ILNDs 48 PLNDs	54 (20–74) median, range	pT1: 21 (46) pT2: 19 (41) pT3: 6 (13) pN0: 22 (48) pN1: 9 (20) pN2-3: 15 (33)	G1: 23 (50) G2: 17 (37) G3: 6 (13)	Common iliac (2 cm above bifurcation), Cloquet’s node, bladder, and genitofemoral nerve *	NA	Positive iliac LNM Absent: 2 Present: 5 Negative iliac LNM Absent: 15 Present: 5	NA	No nodal recurrence in negative lymph nodes of packaged LNDs	21 (8–31)
Chipollin et al., BJUI 2019 [11]	Retrospective multicenter. 1980–2017	Identifying an optimal lymph node yield and its prognostic impact	532 total 198 PLND - 106 bilateral - 92 ipsilateral	59 (49–68) median (IQR)	pT1: 158 (29.7) pT2: 237 (44.5) pT3/4: 119 (22.4) pTx: 18 (3.4) pN0: 146 (27.4) pN1/2: 174 (32.7) pN3: 202 (38) pNx: 10 (1.9)	NA	External iliac, internal iliac, and obturator fossa	No 338 (63.5) Yes 112 (21.1) Unknown 82 (15.4)	NA	A-RT 14 (2.6) CHT 95 (17.9) CHT-RT 39 (7.3)	Local 19 (3.6) Regional 66 (12.4) Distant 55 (10.3)	28 (12–68.2)
Li et al., J Cancer Res Clin Oncol 2016 [12]	Retrospective multicenter. 2000–2015	Bilateral PLND	190 total 69 bilateral PLND 121 No-PLND	52.2 ± 12	≤pT1: 11 (15.9) pT2: 45 (65.2) ≥pT3: 11 (16.9) Tx: 2 (2.9) pN2: 22 (31.9) pN3: 47 (68.1)	G1: 28 (40.6) G2: 30 (43.5) G3: 9 (13) Gx: 2 (2.9)	Common iliac, external iliac, internal iliac, obturator fossa, and pelvic floor	NA	PLND 34 (49.3) No-PLND 50 (41.3)	A-CHT 28 (40.6) A-RT 4 (5.8) A-CHT+RT 5 (7.2)	NA	21.5 ± 23.3
Djajadiningrat et al., J Urol 2015 [7]	Retrospective single-center. 2001–2012	Prophylactic PLND	79 total 23 bilateral PLND	66 (60–74) Median (IQR)	T1a: 12(15) T1b: 4 (5) T2: 52 (66) T3: 8 (10) T4: 2 (3) Tis: 1 (1)	Well: 10 (13) Moderately: 46 (58) Poorly: 23 (29)	Common iliac, ilioinguinal nerve, bladder and prostate, and obturator fossa	No 56 (76) Yes 18 (24) Unknown 5 (6)	Overall No: 34 (43) Yes: 45 (57) Tumor-positive No: 5 (26) Yes: 14 (74) Tumor-negative No: 29 (48) Yes: 31 (52)	Pelvic A-RT: 10	NA	59 (40–72) Median (IQR)
Zargar-Shoshtari et al., J Urol 2015 [13]	Retrospective multicenter. 1978–2014	Criteria for bilateral PLND	140 total PLNM 83 bilateral ILND 64 bilateral PLND 15 unilateral PLND	64 (51–71) median, range	pT1-4 N3M0	NA	Internal iliac, external iliac, and obturator fossa	NA	Inguinal ENE Unilateral PLND 10 (67) Bilateral PLND 54 (84) Pelvic ENE Unilateral PLND 4 (30) Bilateral PLND 38 (60)	A-RT: 34 (41) NAC: 27 (33) A-CHT: 11 (13)	Unilateral PLND Local 0 Regional 7 (0.58) Distant 5 (0.42) Bilateral PLND Local 1 (0.03) Regional 13 (0.36) Distant 22 (0.66)	11 (5.5–20.7) median (IQR)
Zargar-Shoshtari et al., World J Urol 2015 [14]	Retrospective multicenter. 1978–2012	Extent of PLND impacts survival	51 total PLND 38 ipsilateral 13 bilateral	Unilateral 64.5 (35.9–82.8) Bilateral 61 (43.5–74.5)	Unilateral 38 pT1: 11 (0.30) pT2: 19 (0.50) pT3: 2 (0.05) pTx: 6 (0.15) Bilateral 13 pT1: 4 (0.31) pT2: 4 (0.31) pT3: 2 (0.17) pTx: 3 (0.23)	NA	Common iliac (either up to above the ureteric crossover or aorta bifurcation level), internal iliac, external iliac, and obturator fossa	NA	Unilateral PLND No: 18 (47) Yes: 20 (53) Bilateral PLND No: 4 (31) Yes: 9 (70)	Unilateral PLND NAC: 5 (0.11) A-CHT: 9 (0.24) A-RT 10: (0.26) NAC+ A-RT: 2 (0.05) Bilateral PLND NAC: 1 (0.08) A-CHT: 9 (0.69) A-RT: 0 NAC+A-RT: 1 (0.08)	NA	13.3 (6.2–35) Median, IQR
Yao et al., J Urol 2020 [8]	Retrospective single-center. 1999–2018	Lymph node mapping in PC patients undergoing PLND	128 total 111 bilateral PLND 17 unilateral PLND	53 (45–61) Median (IQR)	pT1: 33 (25.8) pT2: 61 (47.7) pT3: 22 (17.2) pT4: 12 (9.3) pN0: 7 pN1: 17 pN2: 21 pN3: 83	G1: 43 G2: 55 G3-4: 30	Presacral, obturator fossa, common iliac, internal iliac, and external iliac	NA	Patients 56 (43.8) PLNM with ENE 42.9% PLNM without ENE 45.8%	NA	Local 3 (2.3) Regional 16 (12.5) Distant 12 (9.4)	20.5 (2–81)

**Table 2:** Inguinal and pelvic lymph node status and survival analysis in patients treated for penile cancer.

Author	Inguinal Lymph Node Ratio/Status Positive/Total Inguinal Nodes	Pelvic Lymph Node Ratio/Status Positive/Total Pelvic Nodes	Outcomes	Conclusions
Zhu et al., 2008 [9]	Median total ILNs 11 (7-15) Positive ILNs 1–15 (45.5) 2–2 (6.1) 3–3 (9.1) ≥4–13 (39.4)	Total PLNs according to positive ILNs 1-2–17 ≥3–16 Positive PLNs according to positive ILNs 1-2–2 ≥3–14	-3-year CSS rate for patients with ILNM was 53.1%. -Only 1/16 patients with PLNM remained disease-free within 31 months.	CT and Cloquet´s node are of limited use in predicting PLNM. ILN status, ENE, and p53 expression are significantly associated with PLNM.
Zhu et al., 2009 [10]	Medial inguinal 6 (4–10)–29% LNM Lateral inguinal 6 (3–8)–4% LNM Cloquet´s node 1 (0–3)–5% LNM Median, range	External iliac 4 (2–7)–15% LNM Obturator 6 (3–9)–4% LNM Common iliac 2 (1–5)–2% LNM Median, range	- PPV and NPV of Cloquet´s node for predicting iliac LNM were 80% and 86%, respectively. - External iliac package was most involved region in PLND - Iliac LNM was absent in 13 groin basins with 1–2 positive ILNs and absent ENE.	Extranodal extension is an important predictor for extended lymph node metastasis beyond the medial inguinal package.
Chipollin et al., 2019 [11]	2 (1–4)/15 (10–22) median	2 (1–4)/13 (8–19) median	≥9 (*n* = 148) vs. ≤9 (*n* = 50) PLN 5-year RFS 60.3% vs. 43.2% 5-year DSS 64.2% vs. 47.2% 5-year OS 60.3% vs. 39.8%	LNY to be a significant predictor of outcomes after lymphatic staging for penile SCC.
Li et al., 2016 [12]	NA	Number of LNM Median (range) 1-3: 25 (36.2) ≥4: 10 (14.5) ENE: 34 (49.3) LNM laterality *N*, % Unilateral 31 (44.9) Bilateral 38 (55.1)	Median survival 20.8 mo. - PLND group 1-year DSS 65.7% 3-year DSS 39% - No PLND group 1-year DSS 65.4% 3-year DSS 39.6% No significant difference	Bilateral PLND may improve survival in pN2 patients. Men with pN3 may not benefit from it.
Djajadiningrat et al., 2015 [7]	NA	2 (2–4)/12 (8–17) median (IQR)	5-year CSS in prophylactic PLND was 51% +pN 5-year DSS 17% −pN 5-year DSS 62%	Inguinal ENE, or ≥2 + ILN are predictive of pelvic tumor positivity in patients without evidence of pelvic involvement.
Zargar-Shoshtari et al., 2015 [13]	-Unilateral positive ILN in unilateral PLND 4 (3–11) -Bilateral positive ILN in bilateral PLND 4 (0-12) -64 patients had bilateral ILNM	-Unilateral positive PLN in unilateral PLND 3 (1–21) -Bilateral positive PLN in bilateral PLND 2 (1–19) -16 (25) patients had bilateral PLNM	Overall survival after PLND Median (*p* = 0.10) -Unilateral 10.9 mo. -Bilateral 11.8 mo. Mean (*p* = 0.10) -Unilateral 12.4 mo. -Bilateral 35.9 mo.	Patients with bilateral ILNM treated with a unilateral PLND should be considered for bilateral pelvic lymphadenectomy in presence of 4 or more metastatic inguinal nodes
Zargar-Shoshtari et al., 2015 [14]	Unilateral PLND Positive ILN 3 (1–6) Median, range Bilateral PLND Positive ILN 2 (1–8) Median, range	Unilateral PLND Positive PLN 2 (1–12) Bilateral PLND Positive PLN 2 (1–9) Median (range)	-Median OS was significantly longer in bilateral PLND patients (21.7 vs. 13.1, *p* = 0.051) -CSS higher in bilateral PLND (21.7 vs. 14.4 mo, *p* = 0.26).	Considering additional therapies and multiple PLNM, bilateral PLND was a significant predictor for improved CSS.
Yao et al., 2020 [8]	3 (2–4)/23 (17–30) median IQR	2 (1–4)/18 (10–30) median IQR	Cohort OS: 23 (2–81) median, range OS PLNM patients: 16 (2–42) median, range OS significantly longer in bilateral than unilateral PLND (30 vs. 18, *p* = 0.004)	Optimal PLND may extend to the common iliac artery, including common iliac, external iliac, internal iliac, and obturator LNs.

CSS, cancer-specific survival; CT, computer tomography; DSS, disease-specific survival; ENE, extranodal extension; ILN, inguinal lymph node; ILNM, inguinal lymph node metastasis; LNM, lymph node metastasis; LNR, lymph node ratio; LNY, lymph node yield; NPV, negative predictive value; OS, overall survival; RFS, recurrence-free survival; PLN, pelvic lymph nodes; PLND, pelvic lymph node dissection; PLNM, pelvic lymph node metastasis; PPV, positive predictive value.

## Data Availability

The authors abide to the journal´s ethical guidelines.
